# FTIR Spectroscopy to Reveal Lipid and Protein Changes Induced on Sperm by Capacitation: Bases for an Improvement of Sample Selection in ART

**DOI:** 10.3390/ijms21228659

**Published:** 2020-11-17

**Authors:** Maria Pachetti, Luisa Zupin, Irene Venturin, Elisa Mitri, Rita Boscolo, Francesco D’Amico, Lisa Vaccari, Sergio Crovella, Giuseppe Ricci, Lorella Pascolo

**Affiliations:** 1 Elettra—Sincrotrone Trieste S.C.p.A., SS14—km 163.5, 34149 Trieste, Italy; maria.pachetti@elettra.eu (M.P.); ireneventurin@hotmail.it (I.V.); elisamitri@gmail.com (E.M.); francesco.damico@elettra.eu (F.D.); lisa.vaccari@elettra.eu (L.V.); 2 Department of Physics, University of Trieste, Via Valerio 2, 34143 Trieste, Italy; 3 Institute for Maternal and Child Health, IRCCS Burlo Garofolo, 34137 Trieste, Italy; luisa.zupin@burlo.trieste.it (L.Z.); rita.boscolo@burlo.trieste.it (R.B.); giuseppe.ricci@burlo.trieste.it (G.R.); 4 Department of Biological and Environmental Sciences, College of Arts and Sciences, University of Qatar, P.O. Box 2713 Doha, Qatar; sergio.crovella@burlo.trieste.it; 5 Department of Medical, Surgical, and Health Sciences, University of Trieste, 34149 Trieste, Italy

**Keywords:** sperm, infertility, FTIR, lipids

## Abstract

Although being a crucial step for Assisted Reproduction Technologies (ART) success, to date sperm selection is based only on morphology, motility and concentration characteristics. Considering the many possible alterations, there is a great need for analytical approaches allowing more effective sperm selections. The use of Fourier Transform Infrared (FTIR) may represent an interesting possibility, being able to reveal many macromolecular changes in a single measurement in a nondestructive way. As a proof of concept, in this observational study, we used a FTIR approach to reveal features related to sperm quality and chemical changes promoted by in vitro capacitation. We found indication that α-helix content is increased in capacitated sperm, while high percentages of the β-structures seem to correlate to poor-quality spermatozoa. The most interesting observation was related to the lipid composition, when measured as CH_2_/CH_3_ vibrations (ratio 2853/2870), which resulted in being strongly influenced by capacitation and well correlated with sperm motility. Interestingly, this ratio is higher than 1 in infertile samples, suggesting that motility is related to sperm membranes stiffness and lipid composition. Although further analyses are requested, our results support the concept that FTIR can be proposed as a new smart diagnostic tool for semen quality assessment in ART.

## 1. Introduction

Selection of good quality spermatozoa is of crucial importance for the outcome of Assisted Reproduction Technologies (ARTs) such as in vitro fertilization and intracytoplasmic sperm injection. Indeed, despite the worldwide spread of ARTs, their efficiency has still to be improved, since over years their successful results (i.e., the pregnancy rate) did not change drastically [1]. Actually, a critical point is the quality of spermatozoa, since in an in vivo condition sperm with major capabilities for fertilization and embryo development were selected during their journey through the oviduct [2]; meanwhile in ARTs, morphology and motility, after spermatozoa in vitro capacitation [3], are the sole parameters routinely used for qualitative assessment. The choice of the best capacitated sperm is then performed using an optical microscope, although with this approach subtle biochemical and genomic alterations cannot be disclosed [4].

To note, the risk of fertilizing the oocyte with defective spermatozoa is maximally increased when they come from men with impaired sperm parameters [5], since they have higher incidence of abnormalities, including DNA fragmentation, that could lead to developmental failure and even affect the offspring in the long term [6].

Different biochemical modifications can also contribute to diminishing sperm fertilizing potential. In particular DNA fragmentation is a significant concern in the field of ART, receiving increasing attention [7]. Numerous assays have been developed to monitor cell suffering, DNA damage [8] and, in general, for identifying various biochemical alterations. However, the idea to test all the potential assays that could be useful for selecting the best cells before ART procedures is impractical, since it implies high costs and long evaluation times. More importantly, the limited quantity of samples usually used in ART does not allow multiple evaluations. Therefore, the availability of non-destructive analytical procedures that simultaneously monitor different parameters would be highly advantageous for an effective selection of good quality semen.

Interesting advanced setups of vibrational spectroscopy, mainly Raman and Fourier Transform Infrared (FTIR) spectromicroscopy, that were recently exploited to reach this aim are emerging [9,10,11]. In principle, it has been reported that sperm can be analyzed at the single cell level with Raman spectromicroscopy, revealing precious information on nuclear DNA status, identifying nucleotide bases damage and/or fragmentation [10] and requiring minimal sample preparation. We also recently demonstrated that DNA damage and modifications may be revealed by UV resonant Raman spectroscopy [12], despite the need of highly concentrated and purified nucleic acids [13]. However, Raman approaches are difficult on intact cells, since the laser induces photodamage thus limiting its direct use on ART specimens.

FTIR spectroscopy is the most promising and effective tool in biomedical research, allowing the detection of sample vibrational features without external labels or laborious sample preparation, while providing comprehensive information on sample biochemical composition and macromolecular structure. Performing FTIR spectroscopy on biological materials, the most informative spectral regions are the fingerprint region (900–1350 cm^−1^) for nucleic acids and sugars, the amide I and amide II (amide I/II) region (1450–1700 cm^−1^) for proteins and the region between 2800 and 3500 cm^−1^ attributable to the stretching vibrations of S-H, C-H, N-H and O-H, and carbon skeleton fingerprint vibrations mainly belonging to lipids [14]. We already used FTIR to reveal oxidative stress mediated DNA damage, artificially induced by in vitro Fenton’s reaction on hydrated sperm samples. Although DNA damage interpretation was partially compromised by experimental artifacts, we showed that strong oxidation of nucleic acids can be revealed through spectral changes in the 1150–1000 cm^−1^ infrared region, the signatures of phosphate stretching bands. In addition, under the oxidant condition, the analysis of other infrared spectral regions highlighted signs of lipid peroxidation, protein misfolding and aggregations [9].

To further deepen the potentialities of FTIR spectroscopy for sperm characterization, in the present work we used FTIR spectromicroscopy to evaluate the semen of human male subjects belonging from couples submitted to ART procedures with the aim of unraveling the specific macromolecular changes promoted by the in vitro capacitation process, and the features corresponding to good quality. Moreover, UV-Raman spectroscopy was exploited on sperm isolated DNA in order to reveal nitrogenous bases status.

## 2. Materials and Methods

### 2.1. Sperm Preparation

Six freshly ejaculated semen samples were collected at the Assisted Reproduction Unit of the Institute for Maternal and Child Health IRCCS Burlo Garofolo, Trieste, Italy. Patient samples obtained through masturbation after sexual abstinence (at least two days) were processed according to World Health Organization (WHO) guidelines [4]. All donors signed an informed consent, and the study was conducted in accord with the ethical standards of the Declaration of Helsinki (7th version 2013) and approved by the FVG regional ethical committee (5mille15D1, approval date: 22 October 2019). Sperm were washed in Sydney IVF gamete Buffer (Cook Medical, Bloomington, IN, USA) and an aliquot was prepared by swim-up as previously described [3]. Briefly, 1 mL of semen was washed with a double quantity of medium (Quinn’s Advantage Medium w/HEPES, SAGE BioPharma™, Bedminster, NJ, USA), added with 0.5% human serum albumin (SAGE Assisted Reproduction Products™, CooperSurgical, Trumbull, CT, USA) and then centrifuged at 300× *g* for 10 min. The pellet was resuspended in 0.5 mL of medium in a tube and covered by additional 0.5 mL of medium, gently layered, then the tube was sloped at an angle of 45 degrees and incubated for at least 45 min at 37 °C. At the end, the tube was gently set upright and two aliquots were collected by gentle aspiration with a Pasteur pipette: the upper interface (fraction 1) corresponding to capacitated semen, and the pellet (fraction 3) corresponding to not capacitated cells. Washed base samples are referred to in the paper as the control. Small aliquots of control and fractions 1 and 3 were examined for sperm concentration and sperm motility.

### 2.2. FTIR Spectroscopy

FTIR measurements were carried out at the Chemical and Life Science branch of the infrared Beamline SISSI, Elettra Sincrotrone Trieste (Trieste, Italy), using a Hyperion 3000 Vis–IR microscope (15X condenser/objective) and a MCT (Mercury-Cadmium-Telluride) detector coupled with a Vertex 70 v interferometer (Bruker Optics GmbH, Ettlingen, Germany). Live sperm cells were measured upon washing of the sample and resuspension in NaCl 0.9%. FTIR measurements were taken, exploiting several points of the samples keeping them at room temperature, using a fluidic cell equipped with 0.5 mm CaF_2_ windows, collecting at least 20 spectra (512 scans per each spectrum) with a resolution of 4 cm^−1^. Background spectra were obtained for each fraction on regions where only the buffer is present with the same acquisition parameters. During all measurements, the interferometer was kept in vacuum, while the microscope chassis was purged with nitrogen flow.

### 2.3. FTIR Data Processing and Analysis

Raw IR spectra were corrected for the contribution of atmospheric CO_2_ and water vapor. Additionally, in order to elude artifacts induced by local thickness variations, vector normalization in the entire spectral range was obtained by Atmospheric Compensation and Vector Normalization routines of OPUS 7.5 software (Bruker Optics GmbH, Ettlingen, Germany). Absorbance spectra of hydrated spermatozoa were obtained by subtracting the spectrum of the physiological buffer solution, collected close to the measured cell group. Each sperm spectrum underwent standard vector normalization for comparison purposes. Standard deviation of the average spectra was also calculated. A rubber band baseline was subtracted from the averaged spectra in order to reduce the Mie scattering contribution [15].

Second derivatives of subtracted cell spectra were computed for the average spectra using OPUS 7.5 (Bruker Optics GmbH) in the 3020–920 cm^−1^ spectral regimes (Savitzky-Golay filter, 13 smoothing points). Few spectral regions of interest were selected in order to evaluate possible modifications occurring in lipid content and of protein secondary structure population. Accordingly, the whole spectra were cut into two different spectral regions in order to work with a specific portion such as 3000–2800 cm^−1^ (lipids, proteins) and 1750–1480 cm^−1^ (proteins), respectively. The minima found using the second derivative approach were used as initial values of the Gaussian curves centers used in the fitting procedures, performed by a home-built software called “PlotIR”. The centers of the Gaussian curves were kept free to variate within 4 cm^−1^ around their initial positions, while the Full-Width-Half-Maximum (FWHM) of the curves was let free to variate within 10. The positions of the infrared peaks found in the regions 1750–1480 cm^−1^ and 3000–2800 cm^−1^, respectively, were collected.

### 2.4. UV Resonant Raman (UVRR)

DNA was extracted from the semen samples (base and capacitated sperm) using the salting out method [16] with some modifications. Briefly, about 1–2 millions of cells were lysed with proteinase K (0.15 mg/mL final concentration) in 428 µL of lysis buffer (composed by 10 mM tris, 0.01 M EDTA, 0.1 M NaCl and 3% SDS) for 2 h at 37 °C. Then the samples were precipitated with 224 µL of NaCl 6 M, after centrifugation the nucleic acid were precipitated with 1 mL of cold ethanol and the pellet resuspend after centrifugation and washing in distilled water. Five microliters of DNA solution were deposited by drop casting on an aluminum surface in order to perform UVRR) measurements, carried out at Elettra Synchrotron Radiation facility. A complete description of the experimental apparatus can be found elsewhere [17]. A 244 nm laser source was employed to excite the samples with a power approximately equal to 50 μW. The Raman scattering signal was collected in a backscattering configuration. To avoid photodamaging, samples were continuously shaken during acquisition. The Raman instrument was composed by a Czerny-Turner spectrometer with focal length of 750 mm, a holographic reflection grating of 1800 g/mm and a Peltier-cooled back-thinned CCD. Raman frequencies were calibrated on cyclohexane spectra and the spectral resolution was 5 cm^−1^ [18]. Final spectra were obtained averaging 12 spectra of 30 s, for a total integration time of 360 s.

## 3. Results

### 3.1. Spermiogram

Sperm motility of the capacitated and pellet fractions of the six patients are shown in Table 1. The motility was calculated as the percentage of the total spermatozoa in each fraction that do not remain immobile. To note the patients 2, 5 and 6 had abnormal motility values, being lower of 40%. Other clinical parameters, including volume, concentration, morphology, presence of leukocytes and other cells (as epithelial cells) were in the normal range.

### 3.2. FTIR Analysis

**Figure 1:**
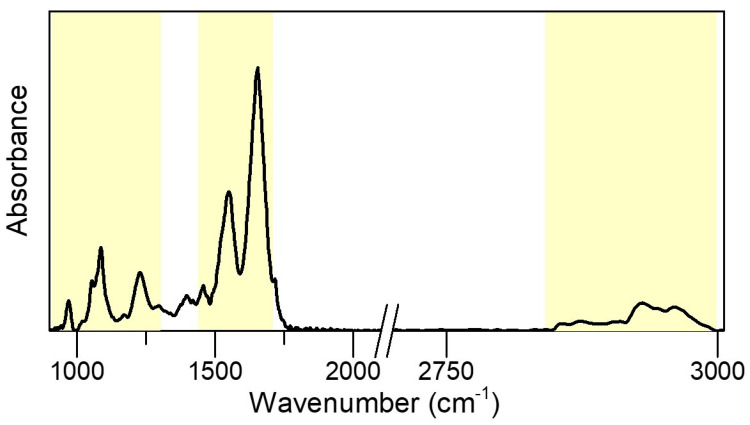
IR spectra: FTIR spectrum of the control sample of patient 4. Colored boxes are used to highlight the spectral regions of interests, such as 1300–900 cm^−1^, that of amide bands (1700—1480 cm^−1^) and, finally, that assigned mainly to lipid vibrations (3000–2800 cm^−1^).

In Figure 1 the FTIR spectrum of a patient sperm cells is presented, where the shadowed boxes highlight the most important spectral regions to be taken into account, namely the region (900–1300 cm^−1^), the amide region (1450–1700 cm^−1^) and the lipid one at higher wavenumbers (2800–3000 cm^−1^). The fingerprint region collects vibrational modes arising from cholesterol, DNA, fatty acids, whereas the other two regions are purely assigned to protein and lipid structures [19]. The FTIR fingerprint region of the different fractions seems to be less sensitive to capacitation () and due to the presence of overlapping bands, we could not quantify whether minor differences between band intensities correlate straightforwardly to spermatozoa motility [20]. For this reason, we decided to focus the study on the regions corresponding to amide bands and lipids. Additionally, possible nucleic acids modifications among the fractions were investigated by means of UV Resonance Raman spectroscopy at 244 nm, an appropriate wavelength to predominantly enhance the nucleic acids contributions avoiding that the spectra were affected by a fluorescence background.

#### 3.2.1. Protein Secondary Structure

Amide bands (1700–1480 cm^−1^) and in particular amide I (1600–1700 cm^−1^) represented the most informative spectral features for the characterization of protein secondary structure. Amide I arose mainly from the C=O stretching of the peptide linkage, and its intrinsic broadness derived from its multicomposed nature. Interestingly, several minima were observed from the second derivative spectra in the amide I region. Each minimum corresponds to a particular protein arrangement in the 3D space. In Table 2 the positions of the sub-bands characterizing amide I are reported with their relative assignment. Despite the major sensitivity of amide I to modifications of protein secondary structures, amide I and II were fitted simultaneously to avoid underestimation of protein subpopulations contents due to the manipulation of the spectra (i.e., its cutting around 1600 cm^−1^).

**Figure 2:**
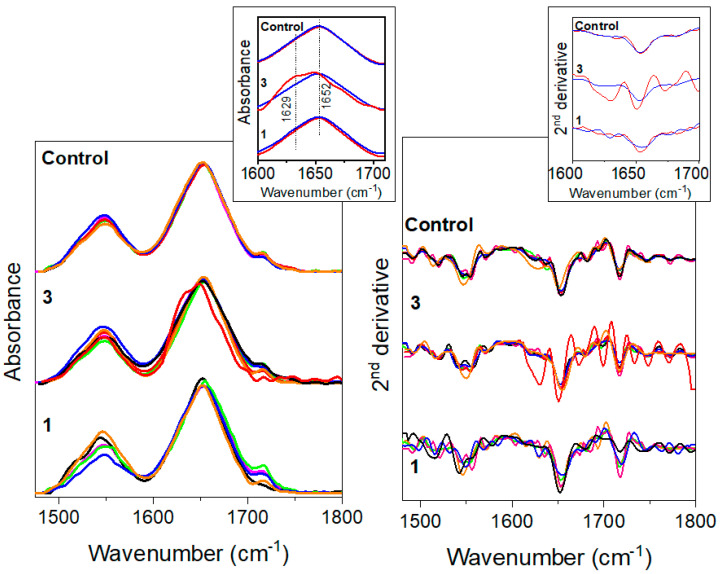
Amide_IR: (left) FTIR spectra and (right) second-derivative of FTIR spectra in the region of amide bands for the control samples (upper part), for fractions 3 (in the middle) and 1 (on the bottom) of the patients considered in this study. Each patient is associated with a specific color: patient 1 is depicted in black, patient 2 in red, patient 3 in blue, patient 4 in pink, patient 5 in orange and patient 6 in green. In the insets, aimed at elucidating the major differences in protein secondary structure composition, we report the findings concerning two cases characterized by important differences in motility, being extreme phenotypes such as very high motility vs. very low motility.

In Figure 2 the amide bands of control, fractions 3 and 1 are reported. Amide I band shape of controls is similar to that of fraction 3, whereas that of fraction 1 is slightly perturbed by the capacitation process. The observed effect must be considered as an average change of the sperm proteome, instead of a specific protein alteration. All the spectra reported in Figure 2 were fitted by Gaussian curves as described in the Materials and Methods, in order to evaluate both the possible secondary structure modification provoked by in-vitro capacitation and, secondly, if we can achieve an average protein composition linked to the motility value.

**Figure 3:**
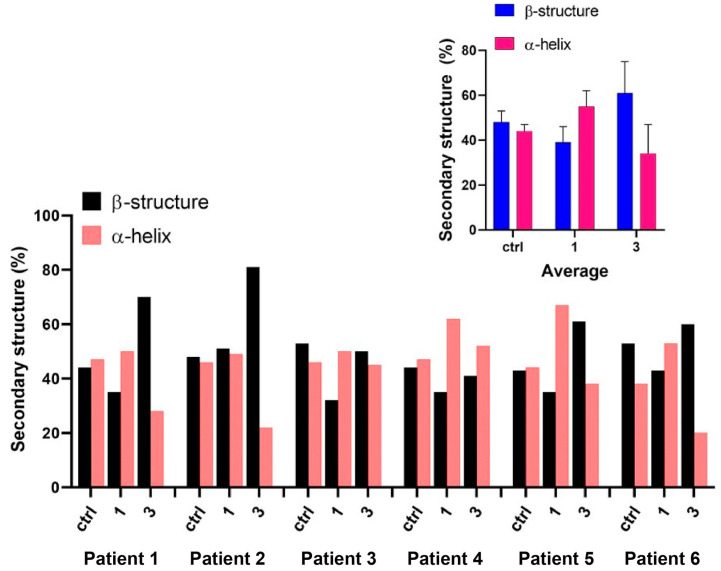
Analysis of secondary structure: percentage of β-structures (in black) and α-helix (in light red) calculated from the fit of the amide region by Gaussian curves, obtained for each fraction considering the 6 patients analyzed. Fraction 1 is mainly characterized by a predominance of the α-helix structure, except for the less fertile patients; differently, fraction 3 is mainly characterized by a massive presence of β-structures. The inset shows the average value and the standard deviation of the secondary structure population percentages among the patients analyzed, showing that fraction 1 can be identifiable by the predominance of α-helix, while fraction 3 of β-structures. Control sample is an intermediate state between the two fractions.

The overall secondary structure composition of each sample is reported in Figure 3. Control samples present a similar content of β-structures and α-helix, with a slight predominance of the former in half the analyzed samples. Fraction 3 samples were actually characterized by the predominance of β-structures, especially in patients 1, 2, 5 and 6. Differently to fraction 3 samples, fraction 1 samples were mainly marked out by a higher content of α-helix in all patients, except patient 2. Thus, the secondary structure variability visible in control and fraction 3 samples was reduced with capacitation, since five over six patients show a markedly enhanced presence of helix-like secondary structure. In the inset of Figure 3 the mean values of both β and α-helix structures are presented with their standard deviation. β-structures and α-helix mean contents in controls samples are 48% ± 5% and 44% ± 3%, respectively, whereas they reach 61% ± 14% and 34% ± 13% in fraction 3 samples. Capacitation overturns these percentages in fraction 1 samples to 39% ± 7% and 55% ± 7%, respectively.

#### 3.2.2. Oxidative Stress: Lipid Peroxidation

In order to observe if the extent of lipid peroxidation could be considered as an alternative evidence of spermatozoa mobility, we focused on the infrared region (3000–2800) cm^−1^, which is mainly characterized by lipids vibrations [14], especially CH_2_ and CH_3_ symmetric and asymmetric stretching. A detailed assignment of the band is reported in Table 3 (lipid).

**Figure 4:**
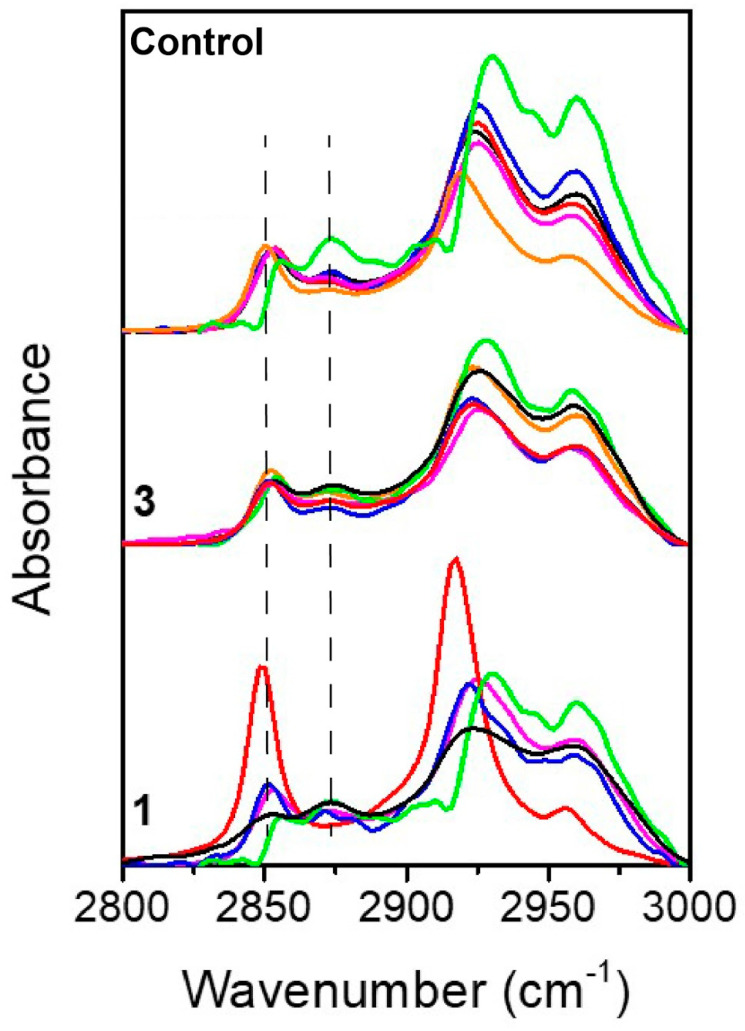
Lipids: FTIR spectra of the lipid region in the region 2800–3000 cm^−1^. In the upper part of the graph, the control samples are shown, then the fraction 3 spectra in the middle and, finally, on the bottom the fraction 1 spectra. Each patient is associated with a specific color: patient 1 is depicted in black, patient 2 in red, patient 3 in blue, patient 4 in pink, patient 5 in orange and patient 6 in green. The two dashed lines are used to indicate the 2853 cm^−1^ and the 2870 cm^−1^ peaks considered for the analysis.

As reported in Figure 4, the shape of this region was maintained throughout spermatozoa capacitation, but a deepened analysis revealed that the relative intensity between two bands at 2853 cm^−1^ and 2870 cm^−1^ resulted influenced by the capacitation process. The 2853 cm^−1^ band corresponded to the symmetric vibration of CH_2_, while the 2870 cm^−1^ band corresponded to the antisymmetric vibration of CH_3_. Interestingly, the former was considered as a marker of lipidic character of a cellular compartment, while the ratio 2853/2870 cm^−1^ was linked to the extent of lipid peroxidation or, more in general, to biophysical modifications of lipidic chains composing cellular membranes [29,30]. In particular, the increasing of this ratio has been related to a higher acyl chain unsaturation level [31].

Similarly, by fitting the whole spectral region by a sum of Gaussian curves, we could calculate the ratio R: R = A(2853)/A(2870) as the ratio of the areas of the abovementioned bands.

**Figure 5:**
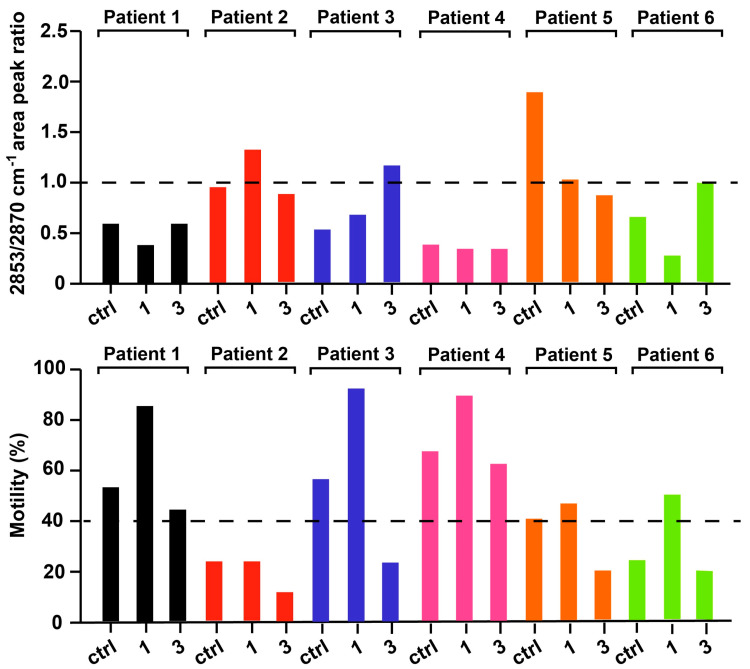
Lipid ratio: the 2853/2870 cm^−1^ area peak ratio (upper panel) and the motility (lower panel) of 6 patients. Each patient is associated with a specific color: patient 1 is depicted in black, patient 2 in red, patient 3 in blue, patient 4 in pink, patient 5 in orange and patient 6 in green. Horizontal dashed lines are used to mark when the area peak ratio between the 2853 and of 2870 cm^−1^ bands is equal to 1 and, in the lower panel, when the motility is 40%, which is considered as a threshold of fertility.

In Figure 5 the R ratios calculated for control, fraction 1 and 3 samples of all the patients are presented. The R value was strongly affected by capacitation and an increase of the ratio was observed in patients with low motile spermatozoa. In fact, almost all the fractions 3 had low motility with respect to the corresponding sample fraction 2 and an R value higher or close to 1. Where the sample was characterized by low motile spermatozoa and the artificial capacitation failed (for example in the case of patients 2), all the fractions analyzed had a R value close or higher than 1. Differently, patients 1, 3 and 4 were characterized by higher motile spermatozoa also in the control fraction and at least fractions control and 1 had a R value lower than 1. The effectiveness of capacitation was clearly manifested in those cases, where low motile spermatozoa were separated from the rest, remaining in the fraction 3 with an R value higher or close to 1. Additionally, when the mobility of spermatozoa in the control fraction was near 40% (patients 5 and 6) and the capacitation was able to select only the high motile ones, fraction 1 had a R value lower than 1, whereas fraction 3 had a R value close to 1.

Thus, on the basis of the R value (i.e., to the extent of lipid peroxidation), we could distinguish fractions that were characterized by spermatozoa with low motility from those that had good quality values.

**Figure 6:**
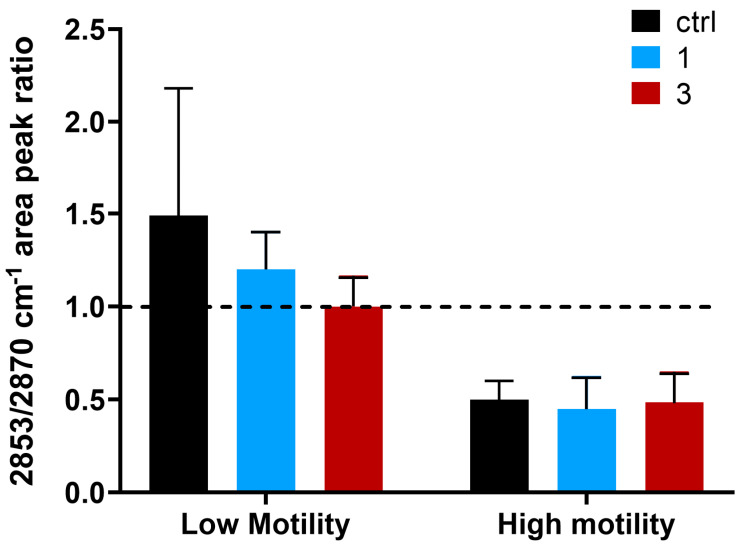
Average_R value: the average value of the 2853/2870 cm^−1^ area peak ratio of the control samples, of fractions 1 and 3 of patients with high (on the right) and low motility (on the left; i.e., motility higher or lower than 40%). Control samples are depicted in black, while fractions 1 and 3 in light blue and red. The standard deviation is indicated in black over the bars. As well-reported in the graph, each ratio reported in the graph is the average value obtained choosing the patients’ fractions with a motility lower (on the left) or higher (on the right) than 40%. In particular, all the patients’ fractions with a measured motility lower than 40% have a ratio higher or close to 1, whereas the average value of the fractions with a motility higher than 40% have a ratio lower than 1.

The results of our analysis are summarized in Figure 6. Control, fraction 1 and 3 samples that had lower motility were characterized by a R value higher or close to 1. In particular, low motile samples (i.e., of patients 2 and 5 of figure motility) had an average R value of control, fraction 3 and 1 of 1.5 ± 0.7, 1 ± 0.1 and 1.2 ± 0.2, respectively. On the contrary, control, fraction 1 and 3 samples that had high motility were characterized by an R value lower than 1. In particular, control samples with high motility (i.e., of patients 1, 3 and 4) had an average R value of 0.5 ± 0.1; fraction 3 samples of 0.5 ± 0.2 and fraction 1 samples of 0.5 ± 0.2.

### 3.3. Quantification of DNA Damaging by UV-RAMAN

**Figure 7:**
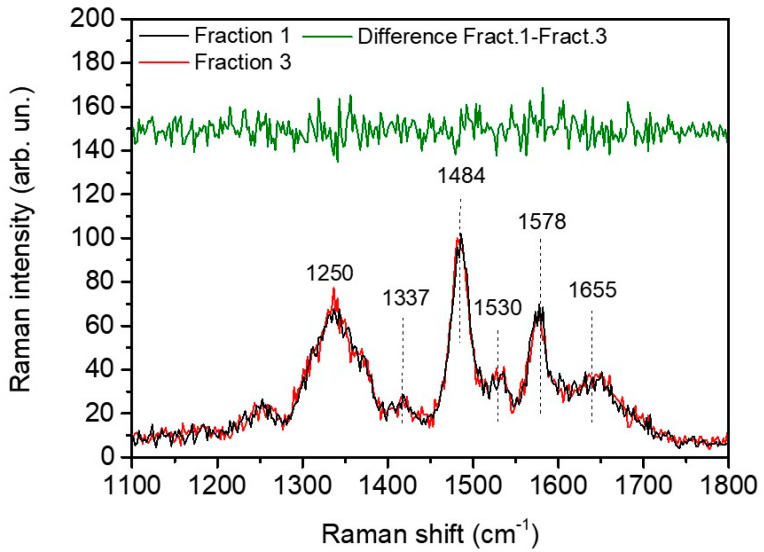
UVRR: UVRR spectra of fraction 1 (black line) and 3 (red line) of patient 3 in the region 1100–1800 cm^−1^. Dashed lines indicate the main peak characterizing the spectra. No significant spectral modifications are visible at nitrogenous bases level. Green line represents the difference spectrum between fraction 1 and 3, highlighting no remarkable difference in DNA damage between capacitated and non-capacitated sample.

DNA vibrations strongly absorb in the region lying within 1250–920 cm^−1^ and, generally, the increasing of the 1013–1080 cm^−1^-bands relative intensity has been reported as a good marker of DNA gradual damaging [32]. However, the overlap between DNA-related bands with amide III band and those addressed to carbohydrates and cholesterol make difficult the disentanglement between those contributions in FTIR spectra. To overcome these limitations, we took advantage of the use of UV Resonant Raman spectroscopy with the aim to selectively enhance the vibrational signals arisen from nitrogenous bases. This choice permits to enhance selectively only the DNA contribution, especially by adenine and guanine, which completely hides those of proteins and lipids. In Figure 7, it is shown the UVRR spectra of control and fraction 1 samples. Both spectra were characterized by the typical line shape associated with nitrogenous bases normal modes. More specifically, the vibration at 1655 cm^−1^ was due to the thymidine C=O stretching, the ones at 1578 cm^−1^ derived from both the adenosine NH_2_ scissoring vibrational mode and from guanosine NH_2_ scissoring and ring vibrations. Additionally, adenosine and guanosine internal ring vibration gives rise to a peak at 1484 cm^−1^ and at 1337 cm^−1^. Finally, a small component at approximately 1250 cm^−1^ can be assigned to adenine, guanine and thymine internal ring vibrations combined with N-H bending. Finally the peak near 1530 cm^−1^ results in a cytosine contribution [33]. From Figure 7 we can see that UVRR did not detect any modifications of the chemical conformations of the nitrogenous bases upon in-vitro capacitation.

In addition, the analyses did not reveal any significant variation at nitrogenous bases level in all the samples.

## 4. Discussion

In ART laboratories the procedures of spermatozoa sample preparation should be simple and economic in order to fit in the routines, and at the same time they must ensure a quick selection of a good number of high-quality cells.

In physiologic condition, spermatozoa become hyperactivated and capacitated during their journey towards the oocytes within the women’s reproductive tract. Approaching the oocyte, the spermatozoa prime and modify their plasma membrane to initiate the acrosome reaction. The capacitation of sperm is achieved through different factors, such as the presence of women cervical mucus, which is rich in capacitation factors, and the interaction with Fallopian tubes epithelium [34]. Lipid membrane modifications during spermatozoa maturation and capacitation is one of the first changes that occurs, and it is characterized by the removement of cholesterol and desmosterol from the surface by protein acceptor molecules like albumin, high-density lipoproteins and apolipoproteins. Intriguingly, cholesterol is a decapacitation factor and is required in order to stabilize the membrane during their journey and to avoid the interactions responsible for the sperm maturation. A low level of oxidative stress is needed for cholesterol efflux [35]. Nevertheless, a high level of oxidative stress, lipid peroxidation and abnormal distribution of polyunsaturated fatty acids (PUFAs) alongside sperm cells are contributing factors to male infertility since they slow down or inhibits the natural process of sperm maturation within male and female bodies [36,37].

In vitro fertilization techniques reproduce what is occurring alongside women’s reproductive tract, and through sperm preparation techniques, the most motile sperm and capacitated sperm were selected for injection into the oocytes.

In the swim-up procedure, which is one of the most used protocols, the incubation of sperm in a medium supplemented with albumin induces an efflux of cholesterol from membranes and an increase of membrane fluidity [35].

In our study, both basal and capacitated sperm were analyzed by using FTIR and UVRR techniques in order to reveal the vibrational spectral features of capacitation and the potential signatures to be monitored for a proper selection of good quality sperm. After in vitro capacitation of six human sperm samples through swim-up method, two different fractions of cells were analyzed, i.e., the capacitated sperm in the upper interface (designed as fraction 1) and the pellet (designed as fraction 3) representing the not capacitated and low-quality cells.

From FTIR analyses we obtained important clues on the macromolecular changes, mainly lipid and protein modifications, induced by the in vitro capacitation process.

Indeed, as shown in Figure 2 and Figure 3, the shape of the amide I in the spectra revealed a modification in the protein secondary structures of sperm samples with a higher presence of β-structure over the α-helix in the pellet cells, and an opposite change in the capacitated cells (upper fraction). Meanwhile in the basal semen the percentages of α-helix and β-structure were similar.

The increment of β-sheet in the pellet cells of fraction 3 may be expected since this conformation is a sign of protein aggregation [38]. Indeed, a high content of β-sheets proteins in cells and tissues and the conversion from an α-helix rich protein population towards a β-sheets-rich one has been previously used as a hallmark of a pathologic status [31]. In this view, the increase of the β-structures could be correlated to a higher percentage of poor-quality spermatozoa, i.e., low motility and not viable cells.

In addition, to explain the abundance of α-helix in capacitated sperm, it is possible to speculate that this is related to the modifications that spermatozoa underwent during capacitation process, indeed it is widely known that alterations in the expression levels of different proteins occur [39].

Very interestingly, the most important feature that we detected in this work was related to the lipid peroxidation, measured by FTIR as CH_2_/CH_3_ vibrations, that results strongly influenced by capacitation and correlated to sperm motility.

While the shape of the lipid region (3050–2800 cm^−1^) [14] was maintained relatively constant throughout spermatozoa capacitation in the different samples, we decided to analyze the ratio of symmetric CH_2_/CH_3_ vibrations (2853/2870) since the two related peaks appeared the most affected. The 2853 cm^−1^ band corresponded to the symmetric vibration of CH_2_, while the 2870 cm^−1^ band corresponded to the symmetric vibration of CH_3_. As reported extensively in the literature, lipid bands are commonly used to diagnose the presence of a biophysical modification of cell membranes such as lipid peroxidation, modification of lipids chain length, oxidative stress and degree of acyl chain saturation level [29]. Noteworthy, this ratio has been recently used also to monitor the benefic effect of cannabis in the treatment of schizophrenia, resulting in a minor disruption of lipid composition, in a reduction of lipid peroxidation and in an increased lipid membrane renewal rate [31].

Our results confirmed that the extent of sperm cell membrane peroxidation, the lower the better, when calculated from the ratio 2853/2870, can represent a fingerprint of spermatozoa motility, potentially applicable in clinical practice. Noteworthy, the CH_2_/CH_3_ ratio could provide important insights regarding the average length of aliphatic chains composing of lipids and fatty acids. Since this ratio was higher than 1 in infertile sperm cells, it indicates that their membranes were mechanically stiffer and composed by a higher concentration of long chain CH_2_ groups, which suggests a weakening of the cell membrane-skeleton structure [30].

In fact, on the basis of the R value we could separate the sperm samples with low or high percentage of motile cells. Patient samples with an R value higher or close to 1 displayed a low percentage of motile cells and intriguingly in some cases in vitro capacitation was able to recover the R ratio to 1 without significantly increasing motility. Instead, when basal specimens present an R value below 1 and close to 0.5, the ratio was not further improved by capacitation, while the proportion of motile cells in the fractions 1 was highly increased.

Alterations in the lipid profile was indeed previously associated with sperm dysfunction resulting in infertility and previous studies already identified some of the lipids mainly involved [40].

It is generally known that the plasma membrane contains approximately 70% phospholipids, 25% neutral lipids and 5% glycolipids [41]. In sperm the definitive lipid patter is acquired after epididymal maturation and it is mainly constituted by significant levels of PUFAs being linolenic acid, linoleic acid and oleic acid, which are the most present ones. From gas chromatography–mass spectrometry and Percoll-selected spermatozoa experiments, Lenzi and colleagues determined that PUFAs consist of 36–52% of the total fatty acids in sperm cells and normal sperm cells possess a higher content of docosahexaenoic acid (DHA) [42,43]. PUFAs contribute to membrane fluidity and flexibility and are precursors of prostaglandins and leukotrienes. Their reduction and, in particular, the reduction of DHA underlies an alteration in the fatty acid definitive pattern with subsequent infertility. As a matter of fact, when the fatty acids distribution contains low content of PUFAs and DHA and the efflux of cholesterol is not efficient throughout the natural capacitation, sperm are characterized by low motility, low flexibility and fluidity and by an atypical morphology [42,43].

As reported by Aksoy and colleagues, asthenozoospermic and oligozoospermic samples are characterized by an anomalously higher content of monounsaturated fatty acids (MUFAs) and lower amount of PUFAs compared to normozoospermic samples, respectively [44]. In addition, both these pathological conditions are characterized by a higher content of saturated fatty acids (SFA) in sperm cells compared to normozoospermic samples [44]. Similarly, Chen et al. observed lower levels of DHA, as the proportion to total sperm lipid content, and a higher amount of ω-3 and ω-6 fatty acids in capacitated sperm from individuals with oligozoospermia or asthenozoospermia with respect to healthy individuals, being capacitation obtained by the discontinuous Percoll gradient [45]. Additionally, a low level of DHA and a high level of elaidic acid (SFA) are found in patients suffering of varicocele [46]. Furthermore, infertile patients exhibited alterations in stearic and polyunsaturated fatty acids in spermatozoa and seminal plasma [47]. Decrement of DHA was also correlated with low sperm motility in individuals affected by retinitis pigmentosa, which manifest this characteristic also in erythrocytes, suggesting that the overall lipid dyshomeostasis of the patients may strongly impact gametes functionality [48]. These observations are consistent also to the negative repercussion that obesity has on fertility [49].

Thus, from the data reported in literature, we could deduce that a higher content of PUFAs (especially DHA) and a lower amount of SFAs and MUFAs had a positive impact on the fertility of sperm cells.

From a structural point-of-view, SFAs consist in an unbranched structure and their general formula consists in CH_3_(CH_2_)n R-COOH. Differently, MUFAs and PUFAs contain one or more double bonds alongside the lipidic chain, respectively. Noteworthy, a lower amount of CH2 (i.e., of SFAs and MUFAs) and a higher content of double bonds (i.e., of PUFAs) alongside the C-C chains was correlated to a higher motility and fertility of sperm cells as our FTIR and biochemical analysis revealed. These evidence could elucidate why pathological conditions of sperm cells were generally related to a low content of PUFAs and a higher content of saturated CH_2_-rich fatty acids.

Notably lipids can undergo detrimental modifications as those induced by reactive oxidative stress (ROS) causing lipid peroxidation. Despite low levels of ROS actually being crucial for achieving spermatozoa fertilizing capability and for their maturation, for chemotaxis, for the acrosome reaction and for their binding to zona pellucida, excessive levels of ROS produce toxic effects. Even if spermatozoa have developed a sort of defense system based on enzymatic and non-enzymatic antioxidants [50], in several conditions the balance between the levels of ROS and of the antioxidants is not maintained, mainly leading to a loss of spermatozoa mobility. An overexposure of spermatozoa to ROS could result in excess lipid peroxidation, which, in turn, could damage the male germ cells’ DNA [50].

We acknowledge a limitation of our FTIR study related to the unspecific identification of the lipid composition of the samples. Indeed, as a future perspective, a complete metabolomic study, including the lipidome, could better elucidate and complement our findings.

Contrary to what was expected based from our previous work [9], FTIR analysis were insufficient to reveal DNA modifications related to the capacitation process, and significant differences among patients with different sperm motility. The main reason could reside in the high complexity of the fingerprint region (900–1300 cm^−1^), where vibrational modes derived from cholesterol, DNA, fatty acids and carbohydrates were mixed. Moreover, the small number and poor homogeneity of patients prevented the identification of promising features to be linked to motility. In order to assess the presence of major damaging of DNA after capacitation, we performed UVRR analysis on isolated DNA. No modification of the chemical composition of the nitrogenous bases was detected. The analyses here performed were related to a small number of patients, and could not be conclusive, however they are in line with the many evidence that lipids are important in the performance of gametes [40].

In conclusion, although we need a statistically robust experimentation, increasing the number of enrolled patients, in order to clinically validate the use of FTIR, our data indicate that R could be proposed as a promising tool to evaluate the quality of spermatozoa and a potential strategy to screen the semen samples, also in the clinical practice of ART centers.

FTIR spectroscopy has been largely employed for the characterization of several cell types and body fluids, female gametes and semen as well, presenting the advantage of being nondestructive and allowing in vivo cell investigations due to the negligible sample heating induced by IR sources [9,51].

In addition, since compact and handy device based on IR spectroscopy are available, we can expect that FTIR spectroscopy will be introduced soon as a fast, inexpensive and easy-to-use diagnostic tool for semen quality assessment, based on the biochemical information contained in the IR spectra of both sperms and seminal fluid.

## Figures and Tables

**Table 1:** Motility of spermatozoa belonging to the single fractions. Fraction 1 represents the capacitated sample, while fraction 3 accounts for the pellet one. The total is the initial sample.

	Motility (%)		
	Fraction 1	Fraction 3	Total
Patient 1	84	44	53
Patient 2	27	11	27
Patient 3	97	20	55
Patient 4	87	61	64
Patient 5	46	25	41
Patient 6	54	22	27

**Table 2:** Proteins: assignment of amide I band positions to secondary structure.

Assignment	Peak Position (cm^−1^)	Literature
β-sheets (intermolecular, extended)	1615–1628	[19,21,22,23]
β-sheets (intermolecular, short)	1629–1635 1680–1696	[19,21,22,23,24]
α-helix	1650–1656	[19,25]
β-turn/loops	1670–1678	[19,25]

**Table 3:** Lipids: assignment of lipids and proteins peaks in the spectral region 3000–2800 cm^−1^.

Assignment	Peak Position (cm^−1^)	Literature
CH_2_ symmetric stretching	2850–2854	[14,26,27]
CH_3_ symmetric stretching	2870	[14,26,27]
CH_2_ asymmetric stretching	2925	[14,27,28]
CH_3_ asymmetric stretching	2956	[14,26,27]
